# Hidden Dangers of Severe Obstructive Sleep Apnea

**DOI:** 10.7759/cureus.21513

**Published:** 2022-01-23

**Authors:** Waiz Wasey, Neha Wasey, Naila Manahil, Sharefi Saleh, Asiya Mohammed

**Affiliations:** 1 Family and Community Medicine, Southern Illinois University School of Medicine, Springfield, USA; 2 General Practice, Shadan Institute of Medical Sciences, Hyderabad, IND; 3 Family Medicine, Southern Illinois University School of Medicine, Springfield, USA; 4 Family Medicine, Ruth Temple Clinic, Los Angeles, USA; 5 Family Medicine, Southern Illinois University Center for Family and Community Medicine, Springfield, USA

**Keywords:** motor vehicle accident, obstructive sleep apnea (osa), sleep attack, microsleep, syncope

## Abstract

Obstructive sleep apnea (OSA) is a breathing disorder secondary to collapsing upper airways while sleeping. The collapse leads to a significant decrease or a complete cessation of airflow despite an ongoing effort to breathe. OSA leads to poor sleep quantity and quality, which, in turn, causes temporary cognitive impairments. Systematic manifestations of OSA can be seen as hypertension, arrhythmias, heart failure, obesity, and worsening of existing pulmonary or cardiac conditions. Severe untreated OSA also leads to significant sleep deprivation, which may eventually lead to sleep attacks. We present a case of a sleep attack leading to a motor vehicle accident that was presumptively diagnosed as syncope. During hospitalization, workup revealed that the patient had very severe OSA. He was treated with a continuous positive airway pressure device, which improved his daytime sleepiness with no new episodes of sleep attacks.

## Introduction

Obstructive sleep apnea (OSA) is a common yet underdiagnosed sleep disorder. Its prevalence in the general population is estimated to be around 34% in men and 17% in women [1]. This breathing disorder manifests as a decrease or complete cessation of airflow despite efforts to breathe while sleeping. OSA is influenced by risk factors such as male sex, advanced age, race, obesity, use of sedative medications, nasal obstruction, and underlying endocrinological disorders such as acromegaly [2]. Certain surgical procedures such as anterior cervical discectomy and fusion (ACDF) can cause new onset or worsening of existing OSA [3]. OSA still remains a medical challenge, with around 80% of patients remaining undiagnosed or being diagnosed late when they present to the hospital with complications [4].

Severe untreated OSA has been associated with temporary cognitive impairments [5], cerebral vascular accidents [6], pulmonary hypertension, heart failure [7], development of metabolic syndromes, increased blood pressure, arrhythmias, and uncontrolled epilepsy [8]. The disturbance to the sleep quantity and quality from severe OSA leads to sleep deprivation. It has been established that sleep deprivation leads to microsleeps and sleep attacks [9]. Sleep attacks are unintended, sudden sleep episodes. Such attacks have been responsible for work-related and traffic accidents. They may also be misinterpreted as syncopal episodes. Herein we present a case of motor vehicle accident (MVA) that was presumptively diagnosed and worked up as syncope but turned out to be a very severe case of untreated OSA.

## Case presentation

A 58-year-old male with a past medical history of hypertension, chronic obstructive pulmonary disease, atrial fibrillation, and anxiety was evaluated in the Emergency Department (ED) following an MVA. The patient’s partner who was with him in the vehicle described him passing out while driving. The patient stated that he felt his eyes cross and he lost consciousness within seconds. No tonic-clonic episodes were described by the partner. No loss of bowel or bladder control occurred. No post-ictal confusion was reported. The patient denied any associated symptoms such as palpitations, chest pain, or shortness of breath. No slurring of speech, confusion, numbness, or tingling was present prior to the episode. The patient had been in good health preceding the accident.

**Figure 1:**
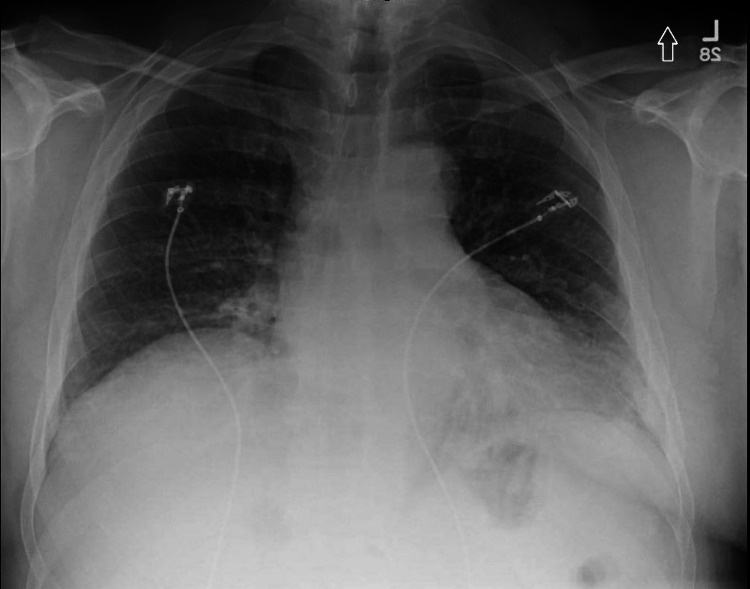
Chest X-ray showing mild left basilar atelectasis and/or infiltrate

Trauma workup in the ED was unremarkable. Mild left basilar atelectasis and/or infiltrate was noted on chest X-ray (Figure 1), but any infectious findings were ruled out with a computed tomography (CT) of the chest. A head CT scan was also negative for hematoma, masses, or other findings that could explain the syncopal episode.

Blood work was negative for significant anemia, hypoglycemia, electrolyte imbalances, or elevated cardiac markers (Table 1).

**Table 1:** Initial laboratory evaluation

Lab Test	Value	Reference Value
Hemoglobin	12.2 gm/dL	14-18 gm/dL
White blood cells	7.2 k/cumm	3.4-9.4 k/cumm
Sodium	140 mmol/L	136-145 mmol/L
Creatinine	0.8 mG/dL	0.6-1.3 mG/dL
Glucose	107 mg/dL	70-105 mg/dL
Troponin	<0.03 ng/mL	<0.03 ng/mL
B-type natriuretic peptide	69 pg/mL	0-72 pg/mL

Urine toxicology was negative for any substance use (Table 2) other than benzodiazepine; this was from a patient being on alprazolam 0.5 mg once a day as needed for anxiety.

**Table 2:** Urine toxicology results

Lab Test	Value
Amphetamine	Negative
Barbiturate	Negative
Benzodiazepine	Unconfirmed positive
Cannabinoids	Negative
Cocaine	Negative
Methadone	Negative
Opiates	Negative
Phencyclidine	Negative
Ethanol	Negative

**Figure 2:**
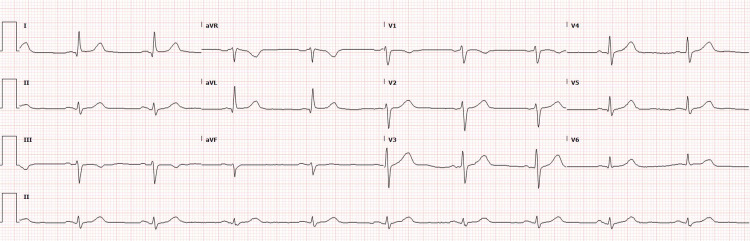
EKG showing sinus bradycardia

Initial electrocardiogram (ECG) performed showed sinus bradycardia (Figure 2).

With no initial significant findings, the patient was admitted for further syncope workup. A cardiology consult did not find a significant cardiac etiology for the episode. Carotid duplex scans reported minimal disease bilaterally with 16-49% stenosis in the internal carotids. Patent flow was seen in the external carotids and vertebral artery flow bilaterally. Echocardiogram revealed an ejection fraction of 64% with normal right ventricular systolic function. Mild aortic valve sclerosis with no significant stenosis was noted as well. The patient was on telemetry during the hospital stay, which did not alert of any arrhythmias or pauses.

**Figure 3:**
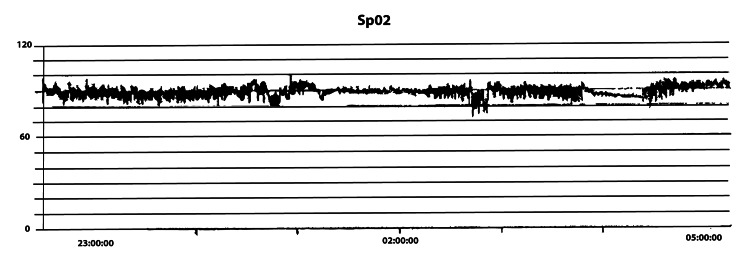
Abnormal overnight oximetry

During clinical rounds, the patient was always found to be asleep with loud snoring. On further interview, the wife reportedly witnessed apnea events and multiple daytime naps. An overnight oximetry was performed in the hospital, which showed an oxygen desaturation index of 24.3/hour, with an oximetry pattern indicating OSA (Figure 3).

Given the significant findings on oximetry along with a history of severe daytime somnolence, the patient was set up for a polysomnography evaluation. On discharge, the patient was also given a 30-day event monitor, which revealed sinus rhythm with no supraventricular tachycardia, atrial fibrillation, AV block, or pauses.

**Figure 4:**
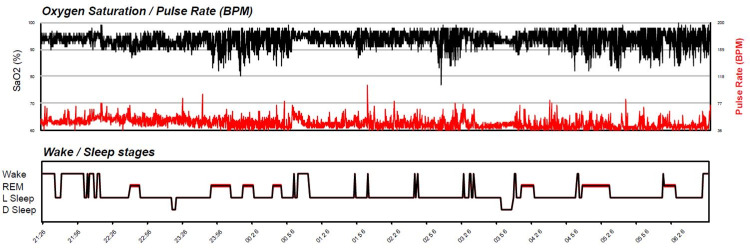
Abnormal home sleep study parameters indicating severe obstructive sleep apnea

Home sleep study revealed very severe OSA with an apnea-hypopnea index (AHI) of 98/hour (Figure 4). The following parameters were noted in the sleep study (Table 3).

**Table 3:** Home sleep study results AHI, apnea-hypopnea index; REM, rapid eye movement

Sleep Study Parameters	Findings
Total sleep time	8 hours 16 minutes
Percentage of REM sleep	16.4 %
AHI	98/hour
Mean O_2_ saturations	93%
Minimum O_2_ saturation	77%

He was then started on an auto continuous positive airway pressure (CPAP) device and was followed up in 45 days at the sleep clinic. The compliance report from the CPAP showed adequate use with a reduced AHI of 3.5, indicating good control of the OSA. Subjectively, the patient also reported significant improvement in the daytime somnolence with no new sleep attack episodes.

## Discussion

OSA is a common yet underdiagnosed sleep disorder [1]. It manifests as a decrease or complete cessation of airflow despite efforts to breathe during sleeping. Severe untreated OSA has been associated with temporary cognitive impairments [5], cerebral vascular accidents [6], pulmonary hypertension, heart failure [7], development of metabolic syndromes, increased blood pressure, arrhythmias, and uncontrolled epilepsy [8].

It has been hypothesized that the presence of microsleep may represent a marker of sleepiness secondary to OSA [10]. Microsleeps have been a major concern for MVAs. Research is being conducted extensively in the automobile industry to detect microsleep in order to avoid accidents [11]. The significant amount of disturbance to the sleep quantity and quality from severe OSA leads to sleep deprivation. It has been established that sleep deprivation leads to microsleeps and sleep attacks [9]. Sleep attacks are unintended, sudden sleep episodes. Such attacks have been responsible for work-related and traffic accidents. They may also be misinterpreted as syncopal episodes.

Given the extensive workup, our patient likely had an episode of microsleep or sleep attack leading to the accident. The major workup for syncope or arrhythmia was negative. The severity of sleep apnea in the patient points toward the likelihood of OSA-induced sleep attack or microsleep.

OSA is treated with positive airway pressure (PAP) devices in the majority of the cases that help maintain patent airways. Less severe OSA may be treated with mandibular advancement device [12], positional therapy [13], and novel eXciteOSA treatment. Since our patient had very severe OSA, PAP therapy was the best approach.

## Conclusions

OSA is a breathing disorder during sleep that may lead to sleep deprivation and daytime exhaustion. Severe OSA may present with episodes of microsleep and sleep attacks, which occur during the daytime and often while performing tasks. Microsleep has been a major concern for work-related as well as traffic accidents. Timely diagnosis and treatment of severe OSA can prevent accident-related incidents. Our case report highlights how a sleep disorder, often ignored and underdiagnosed, can present as syncope. This should prompt healthcare professionals to screen patients with no positive findings explaining syncope, with an overnight oximetry, during their hospitalization.
